# MNBC-ME categorizes viral and plasmid sequences within metagenomes and identifies putative species or plasmid host

**DOI:** 10.1186/s12859-026-06497-x

**Published:** 2026-06-10

**Authors:** Ruipeng Lu, Tim Dumonceaux, Muhammad Anzar, Athanasios Zovoilis, Kym Antonation, Dillon Barker, Cindi Corbett, Celine Nadon, James Robertson, Shannon H. C. Eagle, Oliver Lung, Josip Rudar, Om Surujballi, Gabriel Wajnberg, Chad Laing

**Affiliations:** 1 National Centre for Animal Disease, Canadian Food Inspection Agency, Lethbridge County, AB T1J 5R7 Canada; 2 Saskatoon Research and Development Centre, Agriculture and Agri-Food Canada, Saskatoon, SK S7N 0X2 Canada; 3 Department of Biochemistry and Medical Genetics, University of Manitoba, Winnipeg, MB R3E 0J9 Canada; 4 National Microbiology Laboratory at Winnipeg, Public Health Agency of Canada, Winnipeg, MB R3E 3M4 Canada; 5 National Microbiology Laboratory at Guelph, Public Health Agency of Canada, Guelph, ON N1G 3W4 Canada; 6 National Centre for Foreign Animal Disease, Canadian Food Inspection Agency, Winnipeg, MB R3E 3M4 Canada; 7 Ottawa Animal Health Laboratory, Canadian Food Inspection Agency, Ottawa, ON K2J 4S1 Canada

**Keywords:** metagenome, classification, plasmid, virus, taxonomic

## Abstract

**Background:**

Plasmids and viruses are two types of mobile genetic elements (ME), that rely on host cells to reproduce and propagate themselves. Recently, metagenomics has greatly facilitated the discovery and characterization of new plasmids and viruses, which relies on accurate identification of these reads in metagenomes. Some state-of-the-art tools can identify plasmid or viral reads, while others are able to identify the probable host or source species of these reads. Since the Minimizer-based Naïve Bayes Classifier (MNBC) tool accurately classifies chromosomal and viral reads to the species level, we extended it to develop the MNBC-ME tool that can also identify plasmid reads and their putative host species.

**Results:**

A standard reference- and test-sequence framework using simulated variable-length reads was used to benchmark MNBC-ME with eleven other state-of-the-art tools for ME identification: DeepMicroClass, geNomad, PPR-Meta, viralVerify, Plasmer, PlasClass, PlasX, VIBRANT, DeepVirFinder, HOTSPOT, and MOSTPLAS. MNBC-ME was the most consistent tool at classifying chromosomal, viral and plasmid reads of variable lengths, in contrast to the other tools whose precision or recall dropped below 50% in some circumstances. MNBC-ME also exceeded 65% and 70% performance in predicting host genus and family of plasmid reads, respectively.

**Conclusions:**

MNBC-ME is tool for identification of both short and long viral- and plasmid-originated reads across a wide variety of read types. It also identifies potential low-level host taxa for plasmid reads, and source taxa for chromosomal and viral reads. It is freely available at https://github.com/ComputationalPathogens/MNBC-ME and can be found as the ‘mnbc-me’ package in bioconda.

**Supplementary Information:**

The online version contains supplementary material available at 10.1186/s12859-026-06497-x.

## Introduction

Plasmids and viruses are two types of mobile genetic elements that rely on host cells to reproduce and propagate themselves [2]. Plasmids can enhance virulence and confer antibiotic resistance to bacteria and transmit these traits to other organisms by horizontal gene transfer [27]. Viruses can be fatal to both prokaryotes and eukaryotes, but are also used in bacteriophage therapy to treat bacterial infections in humans [13]. Historically, most known plasmids and viruses were derived from clinical isolates and model species [2]. The PLSDB database [17] contains validated plasmids from the National Center for Biotechnology Information (NCBI) [22] and International Nucleotide Sequence Database Collaboration (INSDC) [3] databases, free from chromosomal and redundant sequences. Virus-Host [16] is a comprehensive and manually curated database of viruses from the NCBI GenBank and RefSeq collections. In recent years, metagenomics has greatly facilitated the discovery and characterization of new plasmids and viruses, which relies on accurate identification of ME-originated reads in metagenomes [2].

Several state-of-the-art tools (DeepMicroClass [9], geNomad [2], PPR-Meta [6], viralVerify [1]) classify reads into three general types: prokaryotic chromosomes, plasmids and viruses. Plasmer [28], PlasClass [18], PlasX [27], separate plasmids from chromosomes based on probability thresholds. VIBRANT [12] and DeepVirFinder [19] identify viral reads. MOSTPLAS [30], and HOTSPOT [11] determine the probable source of identified plasmid sequences.

Machine learning classifiers are used in all these tools to make predictions, whose input features are either the whole read sequence or derived from specific markers. The machine learning classifiers used include neural networks, decision tree ensemble models, transformers, Naïve Bayes classifiers and logistic regression models. The sequence markers used include proteins predicted by Prodigal [10], k-mers, and plasmid- and chromosome-specific motifs. DeepMicroClass and PPR-Meta use bi-path convolutional neural networks directly encoding the whole read as a one-hot matrix. The alignment-free branch of geNomad also uses a neural network encoding the read with an IGLOO architecture [25]. DeepVirFinder and PlasClass respectively use multiple k-mer-based convolutional neural networks and logistic regression models to classify reads of different lengths. PlasX also uses a logistic regression model, whose features include alignments of Prodigal-predicted proteins to the Clusters of Orthologous Groups (COG) [8] and Protein Family (Pfam) databases [5] by hmmsearch [4] and the de novo gene families inferred by MMseqs2 [26]. viralVerify, VIBRANT, MOSTPLAS, Plasmer and the marker-based branch of geNomad also use Prodigal to predict proteins to respectively feed into a Naïve Bayes classifier, neural network, random forest classifier and decision tree ensemble model. HOTSPOT uses protein clusters, plasmid Inc, and MOB/MPF information as input for its transformer-based classifier. Plasmer also uses the percent of shared k-mers, the plasmid-specific *oriT* and chromosome-specific rRNA motifs as additional features. geNomad finally aggregates the outputs of the alignment-free and marker-based branches with a weighted linear model.

Despite identifying general types, these tools were not designed to give the species of chromosomal and viral reads, or the host species of plasmid reads. Tools to assign species to previously identified viral reads include WIsH [7] PHIST [29], and iPHoP [21], among others [23]. Tools to identify the host-source of plasmid reads include the aforementioned MOSTPLAS and HOTSPOT.

The multithreaded Minimizer-based Naïve Bayes Classifier (MNBC) tool [14] accurately classifies chromosomal and viral reads to the species level. In this study we extended its functionality to identify plasmid reads by adding known plasmids to the reference database, and to predict the potential host species of plasmid reads based on the plurality voting of candidate plasmids. We benchmarked it against nine other ME identification tools, and two plasmid host prediction tools.

## Methods

### Implementation of the MNBC tool

The MNBC tool [14] uses the minimizer concept [20] and Naïve Bayes classifier to assign source taxa to chromosomal and viral reads. A minimizer is the lexicographically smallest canonical k-mer in a sequence window of length 2k-1 (i.e. k consecutive k-mers). Suppose that the reference database consists of * S* genomes {$\:{G}_{1}$, $\:{G}_{2}$,…,$\:\:{G}_{S}$}, MNBC builds a database consisting of independent index files, each of which stores the total number of k-mers and hash numbers of unique minimizers in a reference genome. The hash number of a minimizer $\:z=\left[{b}_{k-1}\dots\:{b}_{1}{b}_{0}\right]$ is computed as:

1 $$\:hash\left(z\right)=\:\sum\:_{i=0}^{k-1}map\left({b}_{i}\right)\cdot\:{4}^{i},\:where\:map\left({b}_{i}\right)=\left\{\begin{array}{c}0,\:\:\:\:\:if\:{b}_{i}=A\\\:1,\:\:\:\:\:if\:{b}_{i}=C\\\:2,\:\:\:\:\:if\:{b}_{i}=G\\\:3,\:\:\:\:\:if\:{b}_{i}=T\end{array}\right.$$

Suppose that the query read sequence * R* contains * U* unique minimizers {$\:{z}_{1}$,$\:\:{z}_{2}$,*…*,$\:\:z_{U} {\mathrm{\} }}.$ For each genome $\:{G}_{i}$ ($\:1\le\:i\le\:S$), MNBC computes the ratio of shared $\:R\&{G}_{i}$ minimizers to * U*. $\:{G}_{i}$ is rejected in the case of a ratio smaller than the $\:\mu\:$ parameter. If all * S* genomes are rejected, * R* is identified as an unknown read and labelled as unclassified; otherwise, MNBC computes the score of each retained genome as:

2 $$\:\mathrm{log}P\left(R|{G}_{i}\right)=\sum\:_{j=1}^{U}\left\{\begin{array}{c}\mathrm{log}\frac{1}{count\left({G}_{i}\right)},\:\:\:\:if\:{z}_{j}\:is\:present\:in\:{G}_{i}\\\:\varphi\:,\:\:\:\:\:\:\:\:\:\:\:\:\:\:\:\:\:\:\:\:\:\:\:\:\:if\:{z}_{j}\:is\:absent\:in\:{G}_{i}\end{array}\right.$$

where $\:count\left({G}_{i}\right)$ is the total number of k-mers in $\:{G}_{i}$ and $\:\varphi\:$ is the penalty parameter for read minimizers absent in $\:{G}_{i}$.

Following sorting the scores in descending order, MNBC sequentially computes the difference between adjacent scores, and keeps taking the genomes with the greatest scores as candidates until the first difference between sorted scores exceeds the $\:\theta\:$ parameter. MNBC predicts * R* to originate from the species with the most candidate genomes.

### MNBC-ME extension for identification of plasmid reads

To identify plasmid reads, MNBC-ME also builds the index files of known plasmids and adds them to the reference database. For the read * R*, MNBC-ME also computes the score of each plasmid with Eq. (2), then determines the candidate sequences in the same way as MNBC. If plasmid candidates outnumber both chromosomal and viral ones, * R* is predicted to originate from a plasmid, whose host species is the one with the most plasmid candidates. Otherwise * R* is predicted to originate from the prokaryotic or viral species with the most non-plasmid candidates.

### Performance assessment of MNBC-ME

**Fig. 1:**
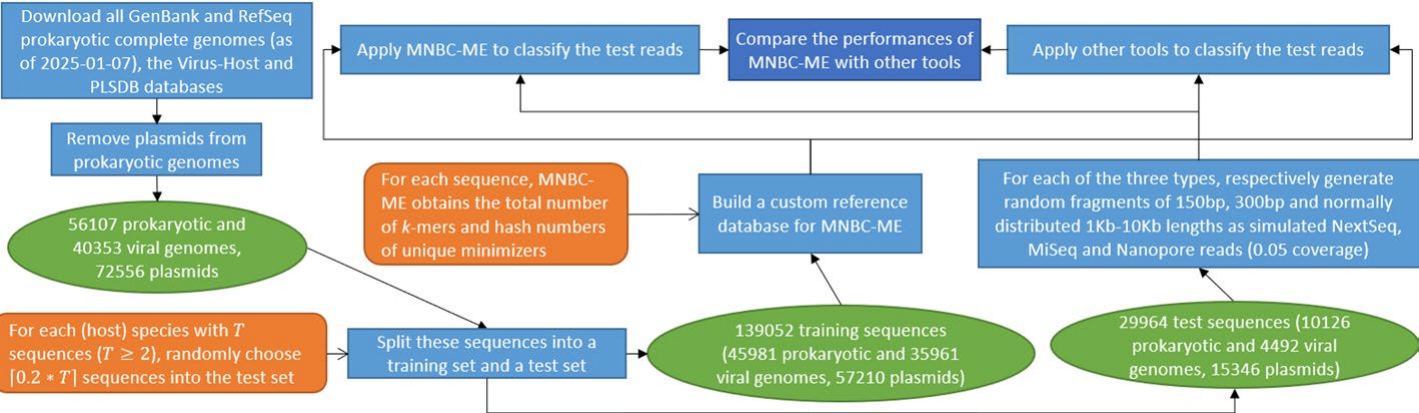
The standard framework to benchmark ME identification tools. All annotated prokaryotic complete genomes excluding atypical ones were downloaded from the NCBI GenBank and RefSeq collections. Plasmids were removed from these genomes as a filtering step. Viral genomes and plasmids were obtained from the Virus-Host database release 227 and PLSDB database version 2024_05_31_v2, respectively. For each prokaryotic and viral species with at least two genomes, 20% of its genomes were randomly picked into a test set. For each host species with at lease two plasmids, 20% of its plasmids were also randomly picked into the test set. All remaining sequences were put into a training set, from which MNBC-ME built a custom reference database. Prokaryotic genomes in the test set were used to respectively generate three sets of random fragments of 150 bp, 300 bp and normally distributed 1–10Kb lengths to simulate reads sequenced by NextSeq, MiSeq and Nanopore technologies. This step was also applied to viral genomes and plasmids in the test set. These test reads were classified by MNBC-ME as well as the other nine tools, then their performance were compared

To assess the performance of MNBC-ME, we developed a standard framework to benchmark it against eleven other state-of-the-art tools (DeepMicroClass v1.0.3, geNomad v1.9.0, PPR-Meta v1.1, viralVerify v1.1, Plasmer v23.04.20, PlasClass v0.1.1, PlasX, VIBRANT v1.2.1, DeepVirFinder v2020.11.21, MOSTPLAS commit 372104, and HOTSPOT v1.1 using simulated sequencing reads (Fig. 1). We only considered tools that did not require assembled contigs as input, and it should be noted that some of these perform better on longer contigs rather than raw reads.

First, we downloaded all GenBank- and RefSeq-annotated prokaryotic complete genomes excluding atypical ones as of 7 January 2025, Release 227 of the Virus-Host database and Version 2024_05_31_v2 of the PLSDB database. Plasmids were removed from the prokaryotic genomes. For each prokaryotic or viral species with T genomes (T ≥ 2), ⌈0.2*T⌉ genomes were randomly chosen into a test set; this was also applied to the plasmids in PLSDB based on the host species. The remaining training genomes and plasmids were used by MNBC-ME to build a custom reference database.

To simulate reads produced by different sequencing platforms (NextSeq, MiSeq and Nanopore), the test sequences were used to respectively generate random sequence fragments of 150 base pairs (bp), 300 bp and normally distributed 1–10 kilobases (Kb) lengths based on 0.05 coverage. 13441246 NextSeq, 6723326 MiSeq and 371870 Nanopore reads were generated from the prokaryotic test genomes; 43381 NextSeq, 23004 MiSeq and 4885 Nanopore reads from the viral genomes; 535249 NextSeq, 271361 MiSeq and 24226 Nanopore reads from the test plasmids. The performance of these tools was measured with both precision and recall, where precision at each taxonomic level = $\:\frac{\#\:reads\:classified\:correctly\:to\:this\:level}{\#\:reads\:classified}$ and recall = $\:\frac{\#\:reads\:classified\:correctly\:to\:this\:level}{\#\:reads}$. In our benchmarking environment, all reads are assigned to only one label, and there are not any unclassified reads. Therefore, the total number of classified reads equals the total number of reads, and precision and recall are identical under this setting. We used the micro-averaging approach to give an optimal per-read consistent evaluation through all tools and taxonomic levels [15, 24].

## Results

### MNBC-ME performance to identify general types

**Fig. 2:**
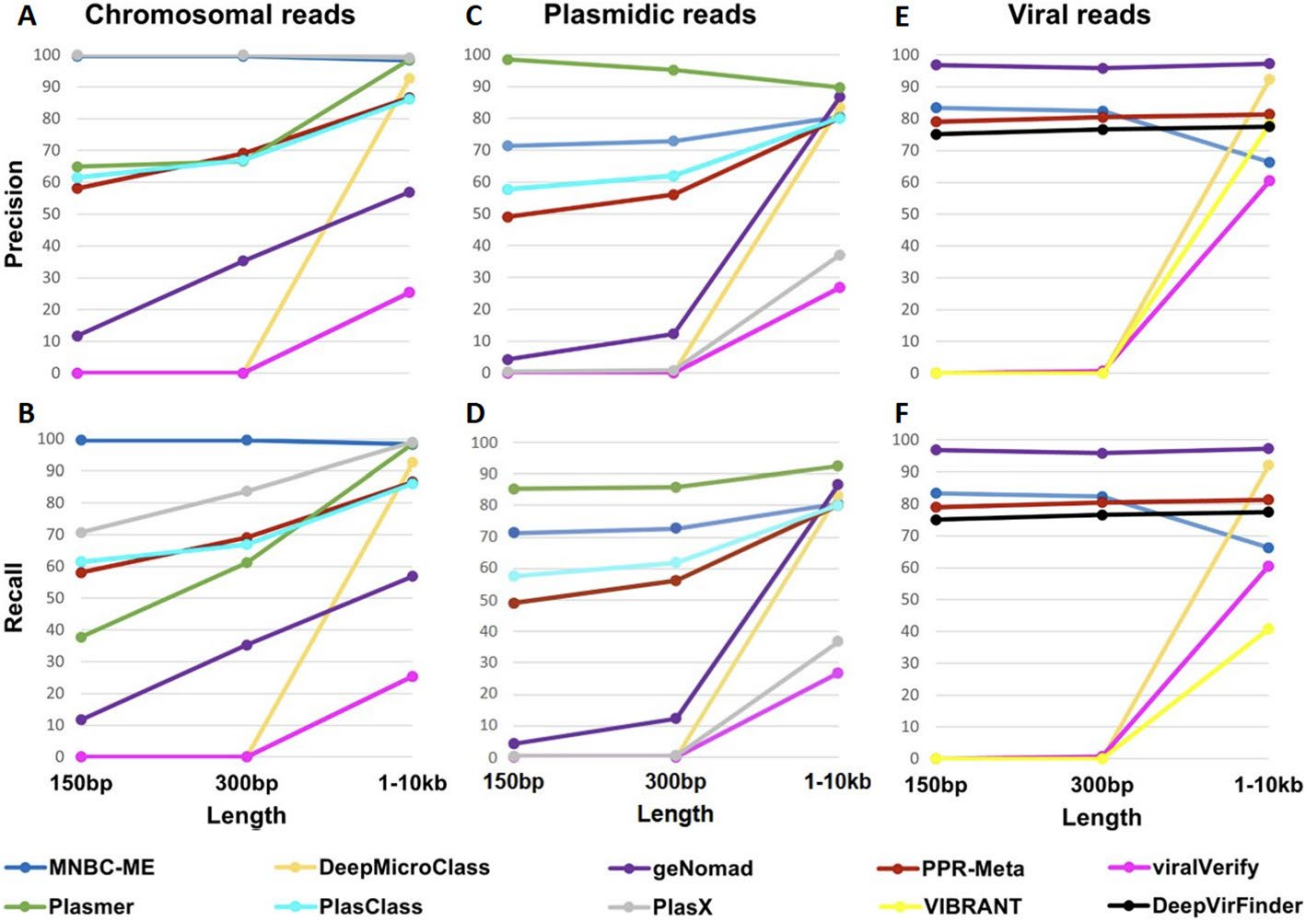
Performance of the ten ME identification tools to identify chromosome, virus, or plasmid categories from the test reads.** A** Precision of eight tools on the chromosomal reads.** B** Recall of eight tools on the chromosomal reads.** C** Precision of eight tools on the plasmid reads.** D** Recall of eight tools on the plasmid reads.** E** Precision of seven tools on the viral reads.** F** Recall of seven tools on the viral reads. MNBC-ME respectively used 0, − 2000, and 1500 as the values of the µ, φ, and θ parameters. The other tools used default parameter values. Overall, MNBC-ME and geNomad performed the best on the chromosomal and viral reads, respectively. MNBC-ME was also the most balanced tool across the short and long plasmid reads. The raw numbers are provided in Additional Table 1

The results of benchmarking MNBC-ME with nine other state-of-the-art tools on the chromosomal, plasmid and viral test reads are shown in Fig. 2.

On the chromosomal reads, PlasX and MNBC-ME exhibited almost perfect precision, but PlasX had a much lower recall on short reads (Fig. 2A and B). Plasmer was on par with PlasX and MNBC-ME on long reads (Fig. 2A and B). Its precision and recall increased as the reads became longer, along with PPR-Meta, PlasClass and geNomad (Fig. 2A and B). PPR-Meta and PlasClass were very close, outperforming geNomad much (Fig. 2A and B).

On the short plasmid reads, Plasmer performed the best, followed by MNBC-ME (Fig. 2C and D). On the long plasmid reads, Plasmer also had the highest precision close to geNomad, DeepMicroClass, MNBC-ME, PPR-Meta and PlasClass, but its recall was much lower (Fig. 2C and D). PlasClass and PPR-Meta also performed closely, outperforming geNomad much (Fig. 2C and D). PlasX had very low performances, close to zero on short reads (Fig. 2C and D).

On the viral reads, geNomad performed the best (Fig. 2E and F). PPR-Meta and DeepVirFinder performed closely, which were exceeded by MNBC-ME on short reads (Fig. 2E and F). VIBRANT could not classify reads shorter than 1 kb, but on long reads its precision was on par with PPR-Meta and DeepVirFinder despite a much lower recall (Fig. 2E and F). It should be noted that among the tools, geNomad also taxonomically classifies viral reads to the family level, and we found classification accuracy of 10%, 22%, and 68% for the simulated NextSeq, MiSeq, and Nanopore reads, respectively.

Overall, DeepMicroClass was not able to classify reads shorter than 500 bp, but performed well on long reads (Fig. 2). viralVerify had close-to-zero performances on all short reads (Fig. 2). It was also a low performer on chromosomal and plasmid long reads (Fig. 2A–D), but improved on viral long reads (Fig. 2E and F).

### MNBC-ME performance to identify low-level taxa

**Fig. 3:**
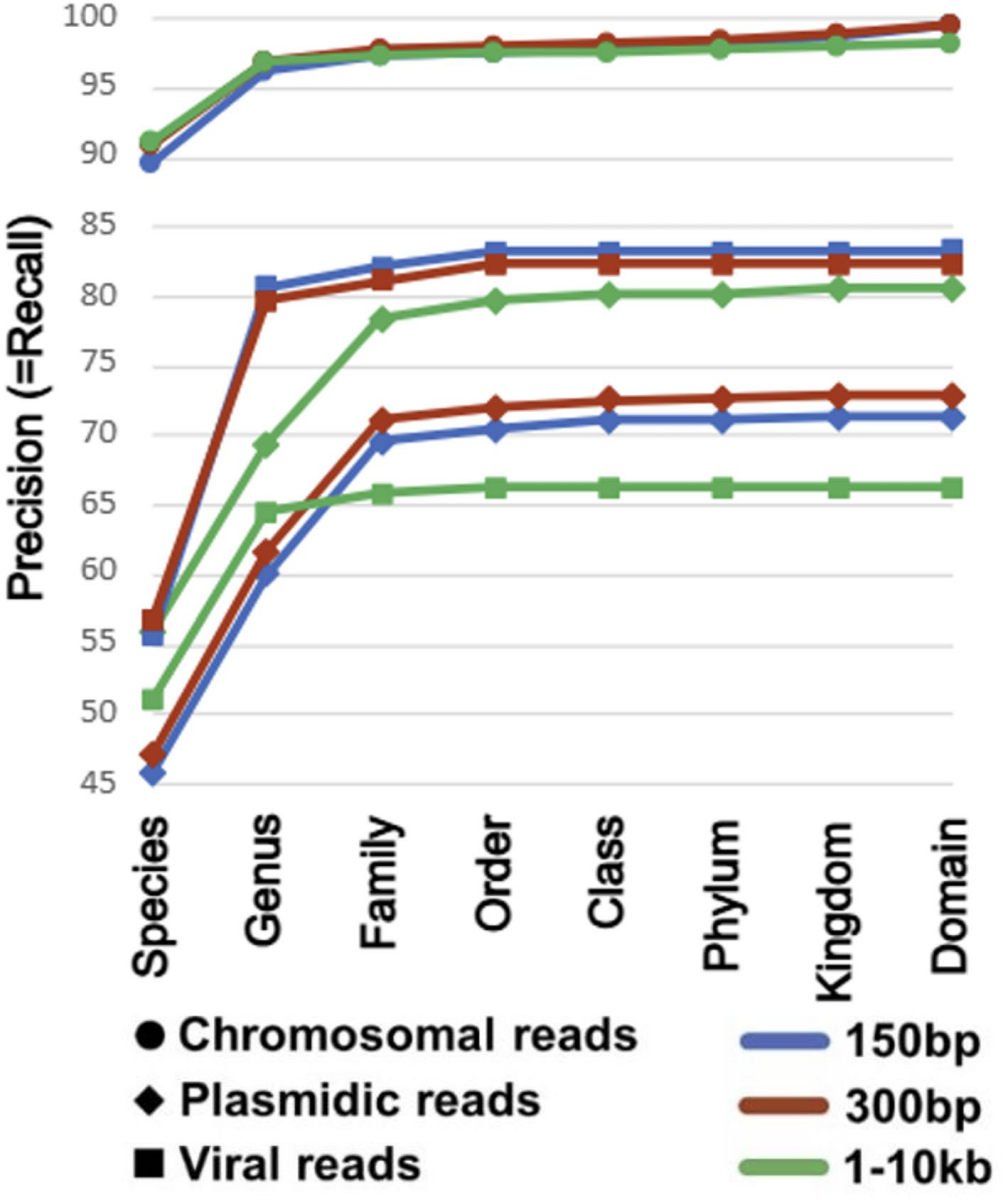
Performance of MNBC-ME to identify low-level taxa of the test reads. MNBC-ME identified taxa of chromosomal reads much more accurately than viral and plasmid ones. Its species-level precision and recall exceeded 50% except on the short plasmid reads. The lowest performance among all reads respectively jumped above 60% and 65% at the genus and family levels, and plateaued at higher levels. The raw numbers are provided in Additional Table 2

In addition to categorizing reads into the three general types, MNBC-ME was also able to further predict lower-level taxa for chromosomal and viral reads, or host taxa for plasmid reads. Its performance at eight major taxonomic levels is shown in Fig. 3. It performed better on the chromosomal reads than on the viral and plasmid ones, achieving about 90% species-level precision and recall (Fig. 3). Species prediction was more accurate on the short viral reads than the long ones, whereas host species prediction was more accurate on the long plasmid reads than the short ones (Fig. 3). A large performance leap occurred on all reads from the species level to the genus level, and also on the plasmid reads from genus to family. As a result, the precision and recall on the short plasmid reads increased from about 45 to 60% then to 70% across the three levels. (Fig. 3).

**Fig. 4:**
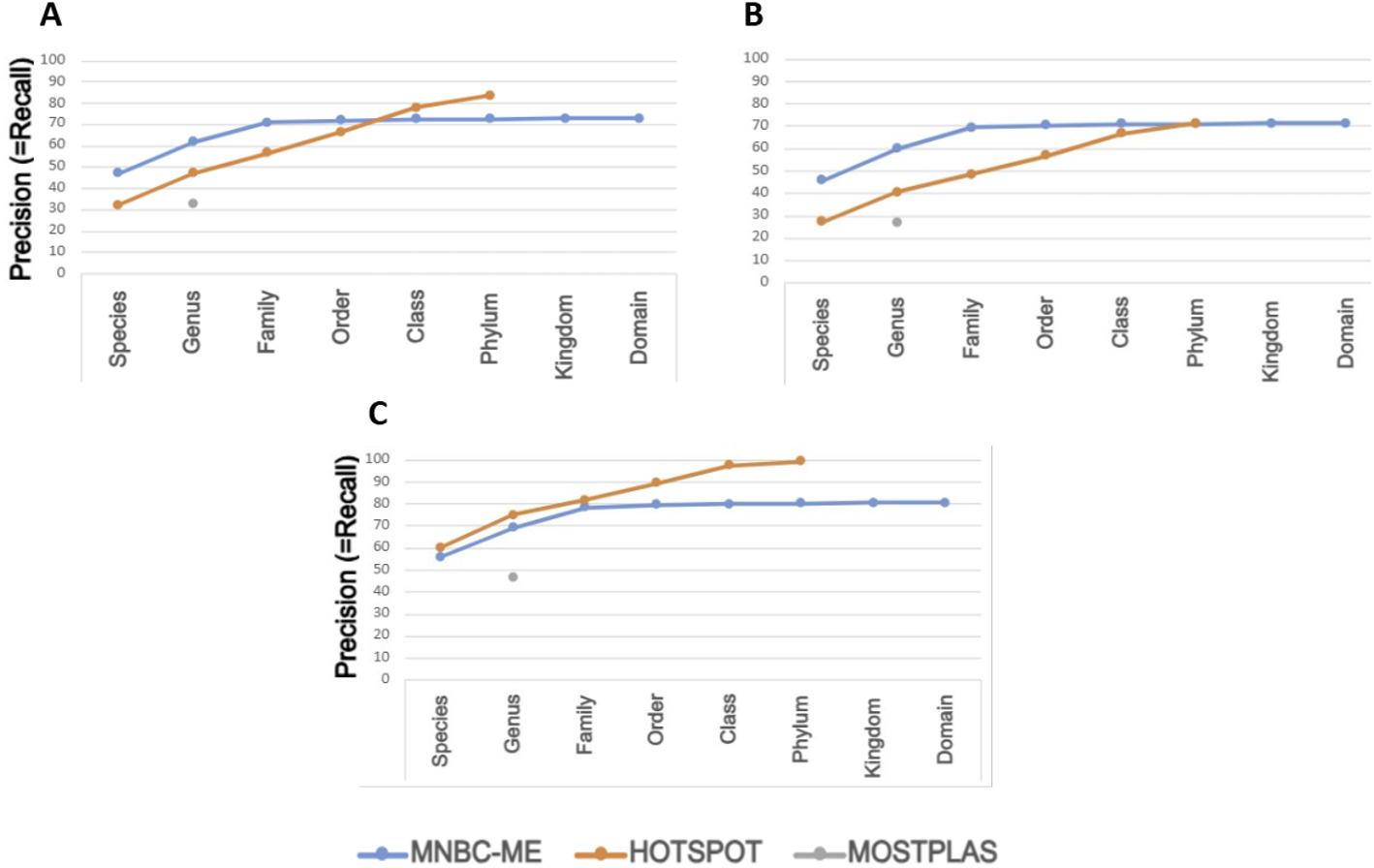
Taxonomic classification of plasmid host, across varying read lengths. Performance of three taxonomic classification tools (MNBC-ME, HOTSPOT, and MOSTPLAS) evaluated across plasmid reads of different sequencing read lengths. Each plot represents Precision (= Recall) for classification at each taxonomic level (x-axis). **A** Precision (= Recall) for plasmid-derived reads of 150 bp,** B** 300 bp, and** C** 1–10 kb. Results are shown across taxonomic ranks from species to domain. MNBC-ME and HOTSPOT are displayed as continuous lines to illustrate trends across taxonomic levels, while MOSTPLAS is shown as an individual point due to its design

To better compare MNBC-ME with tools specifically designed for plasmid host prediction, we examined HOTSPOT and MOSTPLAS using the same groups of reads across the taxonomic levels (Fig. 4). The overall precision (= recall) of MNBC-ME was the highest among these tools at the lowest taxonomic levels, from species to order, for both the 150 bp and 300 bp read sets. For the long-read set, HOTSPOT performed the best, slightly higher than MNBC-ME at all taxonomic levels; MOSTPLAS was specifically designed for genus-level predictions and therefore only has a datapoint at that taxonomic level.

## Discussion

In this study, we took the MNBC algorithm and extended it by adding reference plasmids to its database, to create MNBC-ME that can also identify plasmid reads. All reference sequences in the database were scored and candidates determined in the same way as MNBC. A plasmid read was determined by having more plasmid candidates than chromosomal and viral ones, then plurality voting was used to determine its host species. Otherwise plurality voting of non-plasmid candidates determines the source species of the read, as in the original MNBC program. This contrasts with the other nine benchmarked tools, with the exception of geNomad, that can only predict high-level general types, or the two plasmid-specific host prediction tools we examined.

The benchmarking results of the ten tools on the simulated reads indicated that no tool universally performed the best on all types of reads, though MNBC-ME was good at both read categorization in addition to species or plasmid host classification. PlasX, geNomad and Plasmer were good performers on chromosomal, viral and short plasmid reads, respectively (Fig. 2). However, they deteriorated on other types of reads with precision or recall dropping below 50% (Fig. 2). By contrast, MNBC-ME was more balanced across all three read types, maintaining both precision and recall above 66% (Fig. 2 and Additional Table 1). On low-level taxa prediction, MNBC-ME achieved at least 65% family-level and 60% genus-level precision and recall among all reads (Fig. 2); however, it correctly predicted host species for less than half of the short plasmid reads (Fig. 2). In comparison to the plasmid-specific tools HOTSPOT and MOSTPLAS, MNBC-ME performed best on the 150 bp and 300 bp short-read datasets, while HOTSPOT performed slightly better on the long-read dataset.

In summary, we demonstrated that MNBC-ME is an all-round tool for identifying chromosomal, plasmid and viral reads. It can also report potential source species for chromosomal and viral reads, or host species for plasmid reads, combining ME identification and metagenomic taxonomic classification. Applications of this ability include separating viral and plasmid reads from metagenomes or indicating potential contamination of binned reads or assemblies. As more plasmids and viruses are identified and added to the database, it is expected to become increasingly more accurate due to the plurality voting of candidate sequences. Potential future work includes exploring further improvements to its performance, especially on host species identification of short plasmid reads.

## Supplementary Information

Below is the link to the electronic supplementary material.

Supplementary Material 1.

## Data Availability

The datasets generated and/or analysed during the current study are available in the Zenodo repository, at 10.5281/zenodo.15021745. The prokaryotic genomes (training and test) were obtained from NCBI GenBank and RefSeq, and their sequence accession numbers are in the file prok_training_and_test_sequences_list.txt. The plasmids (training and test) were obtained from PLSDB version 2024_05_31_v2, and their accession numbers are in the file plsdb_training_and_test_sequences_list.txt. The viral genomes (training and test) were obtained from Virus-Host database release 227, and their accession numbers are in the file virus_training_and_test_sequences_list.txt.
